# Boundaries between compulsive buying and hoarding regarding the obsessive-compulsive spectrum

**DOI:** 10.1192/j.eurpsy.2021.1965

**Published:** 2021-08-13

**Authors:** L. Moreno, M. Magalhães, S. Mendes, A. Gamito

**Affiliations:** Department Of Psychiatry And Mental Health, Setúbal Hospital Center, Setúbal, Portugal

**Keywords:** compulsive buying, hoarding, obsessive-compulsive spectrum

## Abstract

**Introduction:**

It has long been theorized that Obsessive-Compulsive Disorder (OCD) and Compulsive Buying Disorder (CBD) may share important characteristics, increasing the likelihood of the cooccurrence of these two psychiatric disorders. On the other hand, Hoarding Disorder (HD) were originally conceptualized to exist only within the context of OCD, despite hoarding symptoms presenting in less than 5% of OCD cases.

**Objectives:**

This study aims to provide an overview of impulsive-compulsive spectrum, regarding the similarities and differential diagnosis between compulsive buying and hoarding.

**Methods:**

The authors performed a non-systematic literature review, using PubMed search terms “compulsive buying”, “hoarding” and “obsessive-compulsive spectrum”.

**Results:**

Obsessive-compulsive spectrum disorders are a group of similar psychiatric disorders characterized by repetitive thoughts, distressing emotions and compulsive behaviors. Compulsive buying is defined by a preoccupation with buying and shopping, by frequent buying episodes or overpowering urges to buy that are experienced as irresistible and senseless. These episodes are accompanied by relief and pleasure, but followed by remorse and guilt. A sub‐group compulsively hoard the items they have bought. Hoarding disorder is characterized by persistent difficulty discarding items regardless of value, urges to save items and distress associated with discarding, and the accumulation of possessions which compromise use of the home.

**Conclusions:**

Empirical evidence suggests that both OCD and CBD display high levels of impulsivity and compulsivity. However, given the phenomenology, CB may not fit well in OCD related disorders. It may be also misleading to classify HD as part of OCD, since hoarding has the lowest specificity and predictive criteria for OCD.

**Disclosure:**

No significant relationships.

